# Circulating, cell-free DNA as a marker for exercise load in intermittent sports

**DOI:** 10.1371/journal.pone.0191915

**Published:** 2018-01-25

**Authors:** Nils Haller, Susanne Helmig, Pascal Taenny, Julian Petry, Sebastian Schmidt, Perikles Simon

**Affiliations:** Department of Sports Medicine, Rehabilitation and Prevention, Johannes Gutenberg-University of Mainz, Mainz, Germany; Sao Paulo State University, BRAZIL

## Abstract

**Background:**

Attempts to establish a biomarker reflecting individual player load in intermittent sports such as football have failed so far. Increases in circulating DNA (cfDNA) have been demonstrated in various endurance sports settings. While it has been proposed that cfDNA could be a suitable marker for player load in intermittent sports, the effects on cfDNA of repeated sprinting as an essential feature in intermittent sports are unknown. For the first time, we assessed both alterations of cfDNA due to repeated maximal sprints and due to a professional football game.

**Methods:**

Nine participants were subjected to a standardised sprint training session with cross-over design of five maximal sprints of 40 meters with either “short” (1 minute) or “long” pauses (5 minutes). Capillary cfDNA and lactate were measured after every sprint and venous cfDNA before and after each series of sprints. Moreover, capillary cfDNA and lactate values were taken in 23 professional football players before and after incremental exercise testing, during the course of a training week at rest (baseline) and in all 17 enrolled players following a season game.

**Results:**

Lactate and venous cfDNA increased more pronounced during “short” compared to “long” (1.4-fold, p = 0.032 and 1.7-fold, p = 0.016) and cfDNA correlated significantly with lactate (r = 0.69; p<0.001). Incremental exercise testing increased cfDNA 7.0-fold (p<0.001). The season game increased cfDNA 22.7-fold (p<0.0001), while lactate showed a 2.0-fold (p = 0.09) increase compared to baseline. Fold-changes in cfDNA correlated with distance covered during game (spearman’s r = 0.87, p = 0.0012), while no correlation between lactate and the tracking data could be found.

**Discussion:**

We show for the first time that cfDNA could be an objective marker for distance covered in elite intermittent sports. In contrast to the potential of more established blood-based markers like IL-6, CK, or CRP, cfDNA shows by far the strongest fold-change and a high correlation with a particular load related aspect in professional football.

## Introduction

Intermittent sports such as football is characterized by repeated sprinting, jogging and walking [1]. While objective tracking allows the assessment of external player load in terms of distance covered, sprints or intense runs, a marker for subjective, internal player load remains to be established [2]. Since tools like rate of perceived exertion (RPE) or questionnaires include the risk of manipulation [3], and efforts to establish molecular biomarkers like creatine kinase (CK), lactate or C reactive Protein (CRP) as markers for exercise load in complex sport settings have failed so far [2–4], such an objective parameter would be meaningful in particular with providing a rational for controlling recovery and optimizing training load.

While cell-free DNA (cfDNA) has initially been shown to increase under acute and chronic pathological conditions like sepsis, stroke, trauma, myocardial infarction, cancer and autoimmune diseases [5], the biomarker increasingly gains importance in exercise physiology [6]. Exercise increased cfDNA levels in blood several-fold after marathon [7, 8], strength training [9, 10] and in laboratory settings including endurance [11] and incremental running [12–14], cycling [15], or rowing exercise [16]. Recently, it was demonstrated that cfDNA increased even during aerobic running below the lactate steady state depending on intensity and duration [17], which points at the enormous potential of cfDNA as a biomarker for exercise load in the aerobic and the anaerobic state.

Therefore, we hypothesize that cfDNA could be applied as a marker for player load in intermittent sports, such as football. The underlying idea is, that cfDNA levels might accumulate or remain elevated over the course of a game, even in periods of moderate jogging or walking [17]. In contrast, other markers like lactate principally increase during anaerobic phases in play, but also decrease during pauses or periods with mostly concentric or aerobic work load [18]. The advantage of cfDNA could be a potential capability of reflecting subjective load of a complete game, since increases occur throughout the game. However, so far, it has not been investigated how cfDNA levels are altered due to a repetitive short bouts of sprints, a characteristic of football in which lactate typically fluctuates around values of 4 to 5 mmol/l [18, 19].

Consequently, we examined for the first time the impact of a standardised sprint training session and moreover, tested for the principal feasibility of cfDNA measurement in professional football during a regular season week including a season game. Firstly, we hypothesise that cfDNA concentrations increase dependent on different intensities in the sprint exercise setting. Secondly, we expect that post-game cfDNA values increase significantly in football players compared to baseline and furthermore correlate with exercise load in terms of distance covered during the game.

## Materials and methods

### Ethical approval

Procedures of the trial were authorized by the Human Ethics Committee Rhineland-Palatinate and conformed to the standards of the Declaration of Helsinki of the World Medical Association. All participants were informed orally and in writing about the experimental setup and the aim of the study and gave written agreement to participate.

### Subjects and exercise setting

Nine healthy subjects were randomly assigned into two groups (A; n = 5 and B; n = 4). After a 15 minute warm-up, the participants performed a series of five sprints of 40 meters each with short (1 minute) or long pauses (5 minutes) between the single runs. To exclude training related variations and a reciprocal influence of both sprint series, we applied a cross-over design in which the participants of group A started with long pauses and group B with short pauses between the sprints. After a rest of 75 minutes, series with different pause times were switched between the groups. The tests were conducted at 8:00 AM. The time of each sprint and the velocity were measured after 10, 20, 30 and 40m with the LAVEG system (JENOPTIK Laser GmbH, Jena, Germany).

For lactate measurement 20μl of capillary blood was collected from the earlobe before and after warm-up, after each sprint and after 3, 15 and 75 minutes of resting following the first and second sprint series. The samples were measured with the Biosen 5130 (EKF Diagnostics, Magdeburg, Germany). At the same time points 20μl of blood from the fingertip was collected to analyse capillary cfDNA. 20ml of EDTA-anticoagulated venous blood was collected from the medial cubital vein after warm-up and immediately after both sprint series. Blood samples taken after warm-up were analysed for complete blood counts.

For cfDNA measurement in football players, exercise testing with cfDNA and lactate analysis was done at the beginning of the season on 23 professional male football players including two goalkeepers in total. Capillary cfDNA from the fingertip was drawn before and after a graded treadmill test until exhaustion. The Test started for every player with a velocity of 6 km/h for 3 minutes and increased by 2 km/h each step [13]. Eight weeks later, we collected baseline cfDNA values from all field players on Monday, Wednesday and Friday during a regular training week ahead of a Saturday game. Following the game, we took capillary blood from those 17 players (3 exchange players, 4 bench players and 10 field players with >70 minutes playing time) who competed in the match. The data from all 10 field players playing more than 70 minutes and for all 4 bench players were analysed. Lactate was measured using plasma from capillary blood samples. Additionally, intense runs, sprints and total distance covered by the players were recorded with the OPTA system (PERFORM Media Deutschland GmbH, Unterhaching, Germany).

### Plasma collection

For analysing capillary cfDNA, blood from the fingertip was centrifuged at 1,600g for 2 minutes at 4°C. Plasma was pipetted and centrifuged at 16,000g for 5 minutes at 4°C to remove cellular debris. Venous blood was centrifuged at 1,600g for 10 minutes at 4°C and plasma supernatant was centrifuged at 16,000g for 5 minutes at 4°C.

### Quantification of cfDNA

Concentrations of nuclear capillary and venous cfDNA were quantified by analysing unpurified plasma via quantitative real-time PCR (qPCR) as described before. In brief, diluted plasma (1:40 in H_2_O) was used as template for qPCR. The amplification was based on primers (5’- TGCCGCAATAAACATACGTG-3’ and 5’-GACCCAGCCATCCCATTAC-3’) targeting a 90bp fragment of human long interspersed nuclear elements (LINEs) of the L1PA2 family. Samples were analysed with a CFX384 Touch™ Real-Time PCR system (Bio-Rad, München, Germany) using the following protocol: 2 minutes incubation at 98°C, followed by 35 cycles of denaturation at 94°C for 10 seconds, annealing at 64°C for 40 seconds and extension at 75°C for 10 seconds [20].

### Statistical analysis

The qPCR data were captured with the CFX Manager 3.0 (Bio-Rad, München, Germany). Microsoft® Excel 2010 was used for data analysis. We considered p-values <0.05 to be statistically significant and performed statistical analysis with JMP8 and JMP13 (SAS, Cary, USA). JMP13 was used for Power-Analysis of cfDNA values. Quantitative changes in parameters at the various time points or differences between pause times were compared by two-factorial analysis of variance (ANOVA). For post-hoc comparisons a Dunnett’s test was used including six time points pre and during exercise using Bonferroni-Holm correction to adjust for multiple comparisons. Non-parametric data were analysed by Wilcoxon and Spearman test, respectively. Correlation of variables was determined by linear regression analysis while the influence of pause time on the variables was analysed by paired t-test. Fold-changes of lactate and cfDNA values in football players during play were determined using the geometric mean of all three resting values set to 1-fold. We had to exclude one single resting value of a player because of physical activity in terms of cycling prior to blood sampling for baseline calculation. Time of blood drawing following the game was monitored. For those players who were more than 10 minutes late for blood sampling after the match (exchange players, players at press conference) on Saturday, values were adjusted for decay of cfDNA as well as lactate based on roughly the same biological half-life of cfDNA and lactate of approximately 15 minutes [13].

## Results

### Sprint interval tests

Anthropometric data of the sprint interval test subjects is given in Table 1. Mean 40 meters sprint time was 5.94 (±0.50) seconds for both series. There was a continuous increase of sprint time within the series of short pauses (+0.15 seconds between the first and fifth run), while sprint time of subjects performing the series of long pauses decreased between the first and third run (-0.10 seconds) and reached baseline time in the fifth run. There were no significant differences in 40 meters sprint time between both series (Wilcoxon test, p = 0.249). In contrast, there was a significant effect of pause time on development of running speed over time. From the third run, velocity development after 30 meters of the athletes performing sprints with short pauses was significantly lower compared to long pauses between the single runs (Wilcoxon test, p = 0.04) This effect was most pronounced after 40 meters (Wilcoxon test, p = 0.005).

**Table 1:** Anthropometric data of the study subjects.

*n*	Sex	Age (years)	Body weight (kg)	Body height (cm)	Training hours per week
9	Whole cohort	23.6 (1.8)	80.9 (11)	179.6 (8.3)	9.9 (3.8)
7 2	Male Female	24.1 (1.6) 21.5 (0.7)	83.6 (10.6) 70.5 (3.5)	181.4 (8.1) 173 (7.1)	10 (4.3) 9.5 (0.7)

Values are given as mean (± SD)

*n* number of subjects

**Fig 1:**
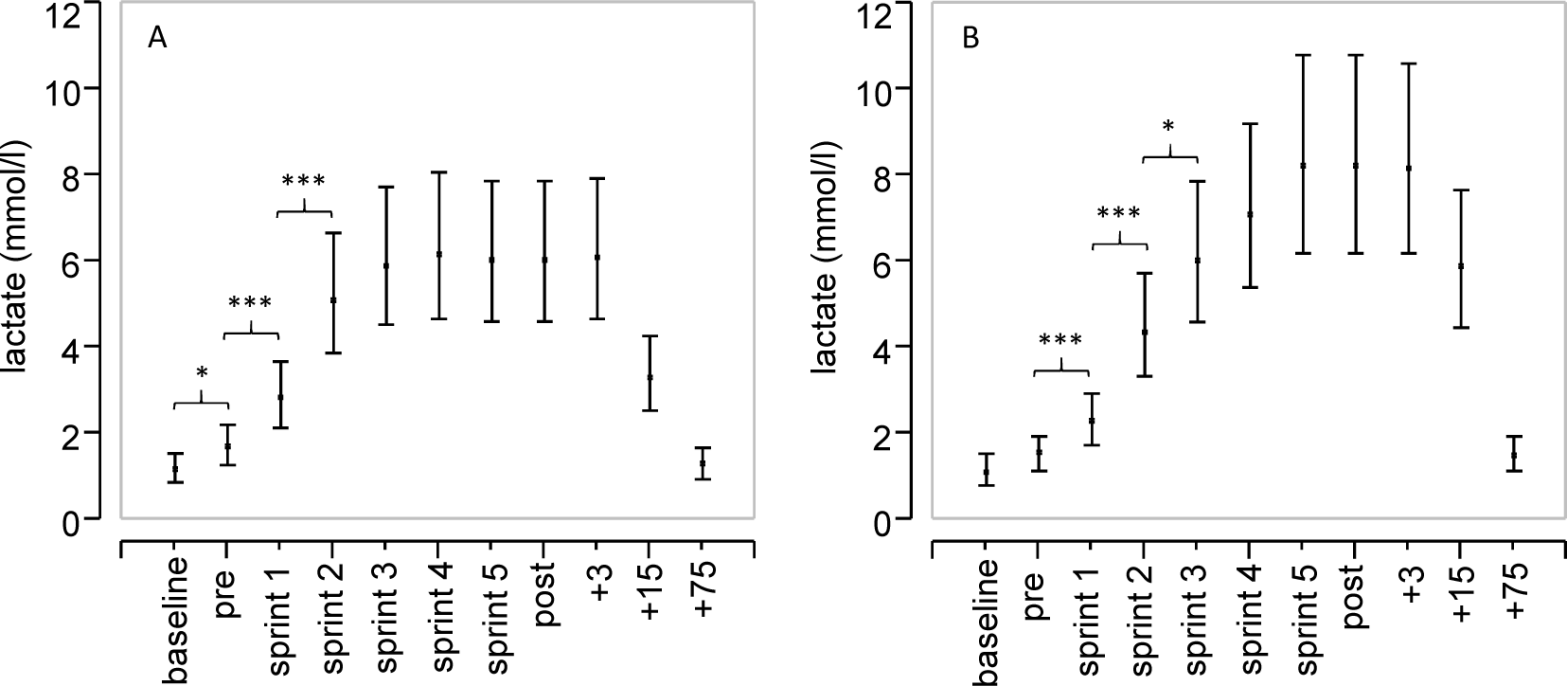
Lactate kinetics during repeated sprint exercise. Concentrations of lactate in athletes before (pre), during (1st– 5th run) and after 3 (+3), 15 (+15) and 75 minutes (+75) of performing five 40 meters sprints with 5 minute pauses (A) and 1 minute pauses (B). Significant difference between both short and long sprint series after the five sprints (p = 0.032). Shown are the mean values and 95% confidence intervals. Significant difference *(p<0.05), ***(p<0.001).

**Fig 2:**
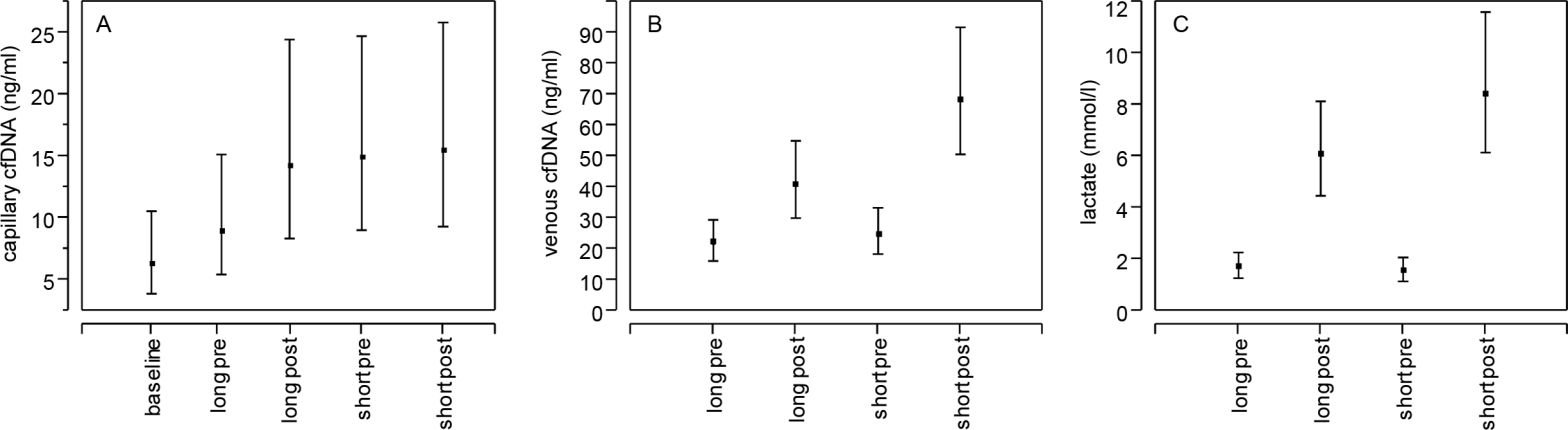
CfDNA and lactate concentrations in sprint series with short and long pauses. Concentrations of capillary cfDNA (A), venous cfDNA (B) and lactate (C) in athletes before and directly after five sprints with 1 minute pauses (short pre and post) and 5 minutes pauses (long pre and post). Significant difference between both sprint series in lactate (p = 0.031) and venous cfDNA (p = 0.016). Shown are the mean values and 95% confidence intervals.

In the setting of long pauses (Fig 1A), lactate reached equilibrium between the third and fifth run, whereas lactate concentrations showed a steady increase due to sprint exercises with short pause times (Fig 1B). Increases from pre to post were significant after both short (5.6-fold, p<0.0001) and long pause (3.6-fold, p<0.0001) series, however lactate values showed a significant difference between both series, reaching 1.4-fold (95%CI: 1.0–1.9; p = 0.032) higher values in the series with one minute pauses (Fig 2B).

Capillary cfDNA values were significantly 1.8-fold (95%CI: 1.1–2.9) elevated following warm-up prior to exercise (p = 0.0168). After warm-up, capillary cfDNA did not show significant alterations (Fig 2A). Concentrations of venous cfDNA increased 1.9-fold (95%CI: 1.2–2.8) due to sprint exercise with long pauses and 2.8-fold (95%CI: 1.8–4.2) after short pauses in between. There was no difference in baseline cfDNA levels between both series. In accordance with lactate, concentrations of venous cfDNA measured after short pause series were significantly higher (1.7-fold, 95%CI: 1.1–2.5) compared to 5 minute pauses (p = 0.016). Venous cfDNA correlated significantly with lactate (r = 0.69; p<0.001).

Power Analysis for venous cfDNA values revealed a power of 1.0 for short pauses and 0.68 for long pauses. A calculation of individual differences between post and pre values revealed a Power of 1.0 for short pauses und 0.95 for long pauses. For capillary samples, we were not able to find mean differences to achieve a sufficient power. While variance of data was not the main problem for lack of an effect, the response pattern of capillary samples seems to be different from venous samples.

### Football tests

**Fig 3:**
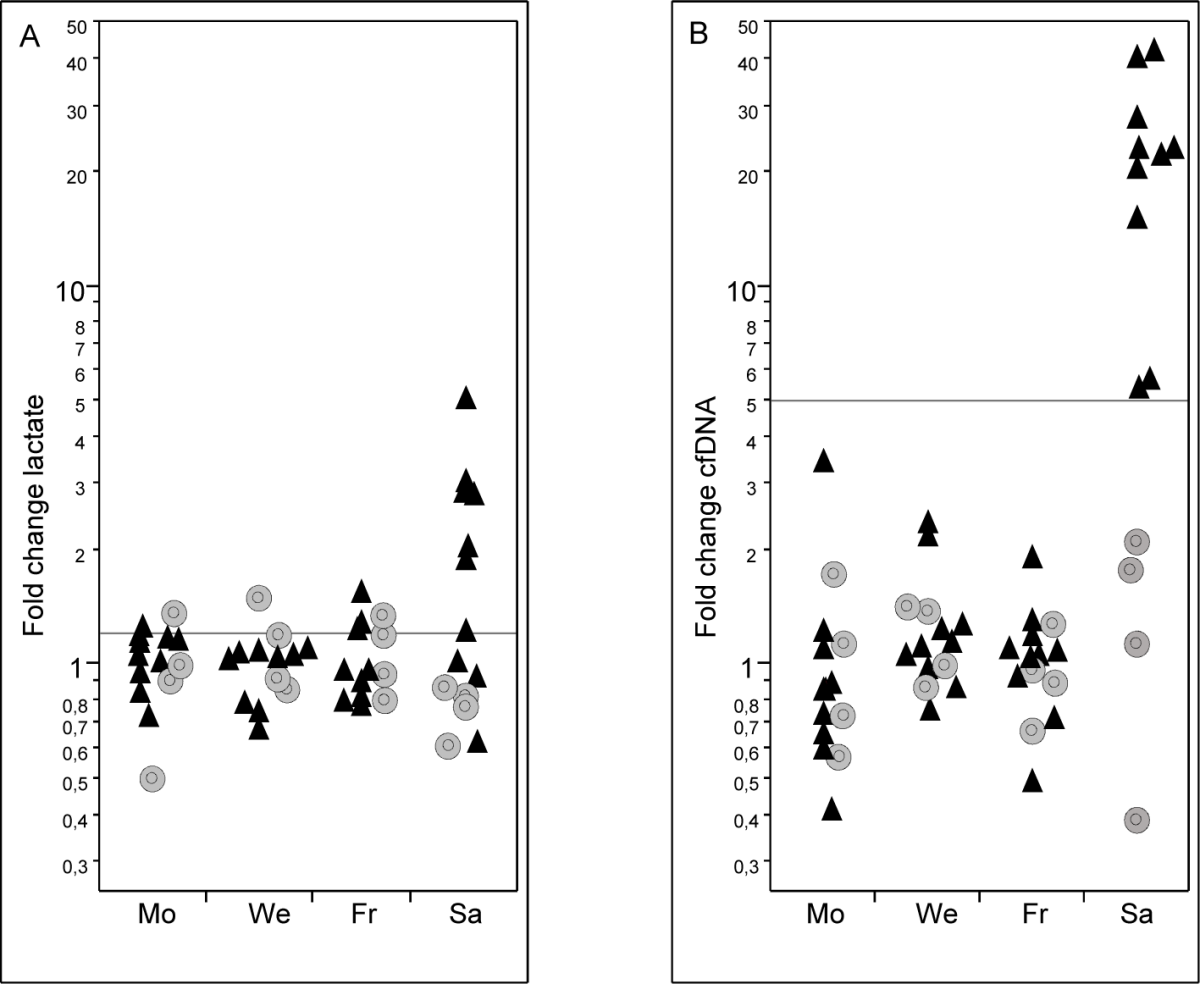
Fold-changes of cfDNA and lactate and after the football game. Fold-changes of lactate (A) and cfDNA (B) of a regular training week at rest (Mo, We, Fr) and post-game values after the match (Sa). Fold-changes were determined using the geometric mean of all three baseline values as 1-fold. Players who played more than 70 minutes on Saturday are in black triangles; bench players in grey circles.

**Fig 4:**
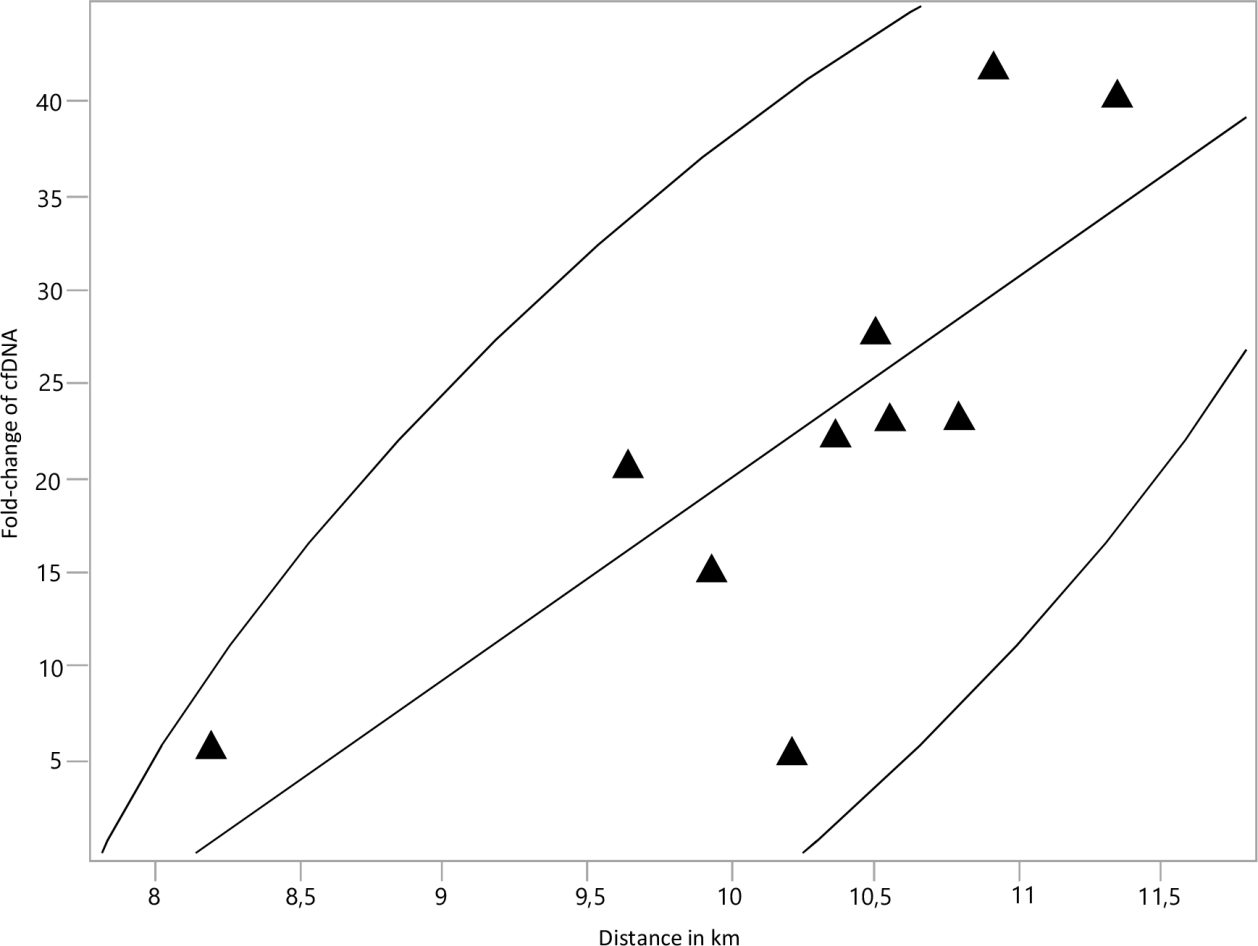
Correlation between total distance covered and fold-changes of cfDNA after the football game.

We took capillary blood samples from 23 male professional football players during a regular season week on Monday, Wednesday and Friday and after the match on Saturday. To allow a comparison between cfDNA and lactate, fold-changes were determined using the geometric mean of all resting values set to 1-fold (Fig 3A and 3B). Blood samples post-game were collected on average after 20.6 (+-9.1) minutes. Median cfDNA increase after correction for decay was 22.7-fold (12.8–30.9-fold; 25^th^-75^th^ percentile) for active players excluding goalkeeper, while lactate relatively increased 2.0-fold (1.0–2.9-fold, 25^th^-75^th^ percentile) after the game. CfDNA increased significantly after the match compared to the mean of the resting values for all players with a playing time of more than 70 minutes (Fig 3B; black triangles; Wilcoxon test, p<0.001). Furthermore, fold-change of cfDNA showed a significant correlation with total distance covered during the game (spearman’s r = 0.87; p = 0.0012, Fig 4), however, no further significant correlation with our tracking data in terms of intense runs or sprints was revealed. In contrast to this finding, fold-changes of lactate showed no statistical significance after the game compared to baseline values (p = 0.09). Moreover, we could not ascertain any significant correlation of lactate with the tracking data. We repeated analysis for cfDNA and lactate increases including only the seven players where we were able to retrieve a sample within 20min following the game without correcting for the decay of the two markers. Even in this conservative setting median cfDNA increased 16.0-fold (8.5–16.8-fold, 25^th^-75^th^ percentile), while lactate values showed a median increase of 0.8-fold (0.6–1.4-fold, 25^th^-75^th^ percentile) compared to baseline.

After step-wise incremental exercise testing, median cfDNA increased 7.0 fold (3.4–10.9-fold, 25th-75th percentile), while lactate increased 10.0-fold (6.1–13.6-fold, 25th-75th percentile). Additional information is given in Table 2, comparing values of those 10 players who competed in the match with the respective values of the exercise test.

**Table 2:** Comparison of cfDNA and lactate after treadmill test and season game.

Type of exercise	n	cfDNA pre (ng/ml)	cfDNA post (ng/ml)	Lactate pre (mmol/l)	Lactate post (mmol/l)
Treadmill test	10	21.9 (8.7)	143.4 (59.3)	1.0 (0.3)	9.6 (2.9)
Season game	10	23.1 (11.9)	396.5 (219.8)	1.7 (0.2)	3.3 (1.8)

Values are given as mean (± SD), Season game pre values are means of resting values.

## Discussion

Many studies addressed the issue to determine player load and to optimize recovery in professional sport settings [2]. Both subjective and objective approaches have shown weaknesses in this regard [2, 4]. Here, we assessed for the first time the response of cfDNA due to repeated sprinting with different pause times and the feasibility of cfDNA measurement during a regular season week in professional football players.

During repeated sprinting, concentrations of venous cfDNA and lactate increased significantly, while kinetics of capillary cfDNA was characterized by a discontinuous increase, which might be attributed to a significant effect of 15 minute warm-up prior to exercise on capillary cfDNA. Absolute concentrations of venous cfDNA were higher than in capillary samples, which is in line with previous findings [13]. In principal, our sprint study revealed that both lactate and venous cfDNA showed higher values in the setting with shorter pause time reflecting a higher level of exertion.

**Fig 5:**
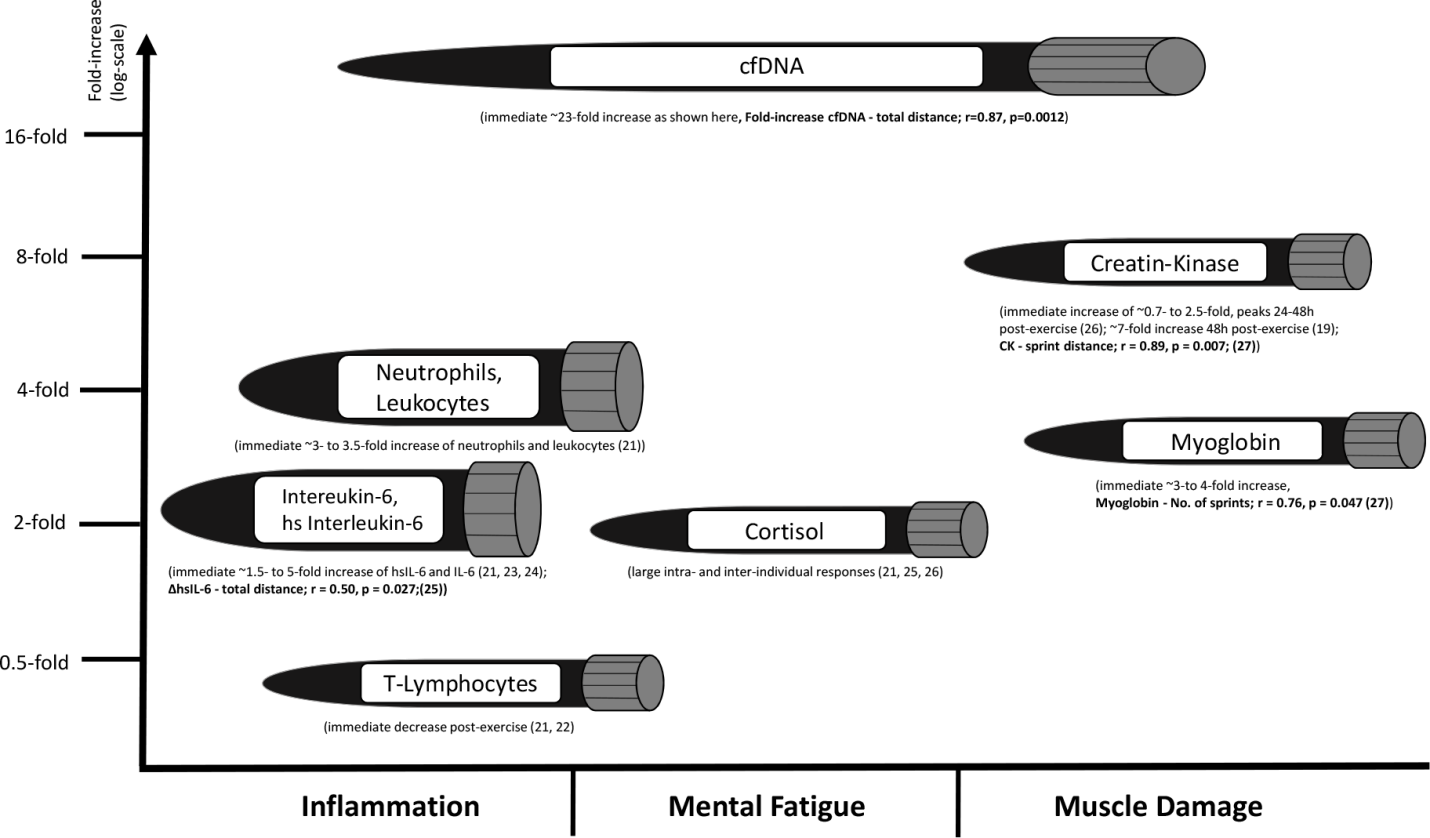
Exercise-induced responses of selected blood parameters due to intermittent exercise. Exercise-induced responses to intermittent sports of selected blood parameters with associated fold-increases and, if already known, the reference to game related aspects in bold.

However, since cfDNA, in contrast to lactate, therefore seem to be capable of reflecting the aerobic as well as the anaerobic state [13, 17], we decided to study the response cfDNA and lactate values due to a professional football match. The outcome was a significant 22.7-fold increase of cfDNA after the game and a high correlation with total distance covered. To our knowledge, this is the most pronounced increase of a biomarker in an acute intermittent exercise setting that has ever been reported (Fig 5) [19, 21–27].

Comparable results to those in the current study were provided by Thorpe et al. [27] and recently by Romagnoli et al. [25] showing that hsIL-6 was correlated with total distance covered in Italian Under-21 players. In this context, cfDNA appears to be a promising approach to determine aspects of player load in intermittent sports such as football.

Clearly, cfDNA requires sophisticated technical equipment and time-consuming measurement; however, qPCR method has advanced in the last few years. The establishing of a direct measurement technique resulted in cost- and time-saving measurement, while a minimum of capillary blood is needed [20]. Moreover, the peak of cfDNA occurs within the first minutes post-exercise [12, 13], while established parameters such as CK peak several days after exercise [1, 19]. RPE is widely used and seems valid for player load, however, includes the risk of manipulation or rather over- or underestimating subjective load [2, 3]. Therefore, a combination of multiple parameters such as heart rate, blood lactate and RPE is recommended, which however seems logistically difficult [28].

Despite the interesting findings in the current study, the mechanism by which cfDNA is released into circulation remains widely unknown. It was shown that cfDNA during exercise originates from the hematopoietic lineage as revealed in a sex- mismatched transplant model. Likewise, in other instances of disease originated cfDNA, there is only minimal literature about the origin so far [29]. Therefore, the value of this paper is limited to the principal feasibility of cfDNA measurement in a (professional) sport setting and its relation to objective performance traits such as running distance. Furthermore, physiological questions have been raised due to our study. It seems that venous values may theoretically be more suitable to reflect repeated sprinting which might have led in the elite sports setting to the fact that only the covered distance during game but not intensive runs or sprinting was associated with capillary cfDNA values.

The source of cfDNA remains speculative, however, a rapid cfDNA accumulation due to active mechanisms seems likely rather than passive cell death events [13]. Beiter et al. suggested neutrophil extracellular traps (NETs) as one source of rapidly released genomic DNA found in blood collected from athletes after 60 minutes of high intensity cycling [30]. The authors suggested that the elevation of cfDNA during exercise is attributed to netting neutrophils releasing their content within seconds. Future studies could aim at demonstrating similarities and differences between oxidative burst of neutrophils and the release of cfDNA during NETosis. Lactate was suggested to be closely associated with the appearance of cfDNA during an incremental treadmill exercise. However, energy expenditure and cardiorespiratory parameters showed a higher correlation with cfDNA indicating that the release of cfDNA into circulation is unlikely to be triggered by lactate [13]. This finding was supported by data showing a continuous increase of cfDNA during 40 minutes of aerobic exercise, while concentrations of lactate remained stable over the course of the run [17]. The results of the present study now even propose a rather indirect association of both parameters. Lactate and cfDNA, particularly in capillary samples, responded differently during the sprint session and also during the football game.

Venous cfDNA concentrations were significantly higher in sprint series with 1 minute pauses compared to 5 minute pauses indicating an either enhanced release of DNA due to higher intensity, or a limited cleavage by DNase I or uptake by cells and subsequent degradation of DNA in the endolysosome via DNase II digestion [17, 31]. Surprisingly, cfDNA did not change significantly following the warm-up in the sprint study. These findings now indicate that immediate release and decay mechanisms at the capillary side could be involved in provoking such difference. Particularly, cfDNA might be taken up by capillary cells and degraded via DNase II in the endolysosome [31]. Moreover, DNase II is a pH-dependent enzyme that is more active at a lower pH-range with an optimum at pH 5. It remains speculative that decreasing pH in the periphery during anaerobic sprinting may have contributed to decreases in intra-endothelial cell pH that might lower endolysosomal pH increasing the potential of the endothelium to cleave cfDNA from the circulation [32–34]. These open questions need to be addressed in more detail by mechanistic in-vivo studies.

To summarize, cfDNA increased in a pause dependent manner during repeated sprinting. Moreover, we assessed a 23-fold increase and a significant correlation with running distance, which points at the potential of cfDNA to reflect load related aspects in professional football. Since this study was conducted to prove feasibility, upcoming investigations should now focus on release mechanisms and reveal the full potential by conducting more standardized intermittent exercise raising additional parameters such as lactate, CK or hsCRP and subjective perceived exertion. Further objective tracking data such as acceleration and deceleration might help with regard at determining subjective intensity during intermittent exercise. It is conceivable that cfDNA analysis in intermittent sports may qualify for two main purposes; 1. determining player load and 2. in a long-term trial to detect overtraining syndrome with respect to previous results [9]. Here, we show that cfDNA due to its enormous sensitivity appears to be a very promising approach.

## Supporting information

S1 File Dataset football 1. Dataset contains data of regular training week and incremental exercise testing.
(XLSX)

S2 File Dataset football 2. Dataset contains data of the football game.
(XLSX)

S3 File Dataset sprint. Dataset contains data of the sprint study.
(XLSX)

## References

[1] Ispirlidis I, Fatouros IG, Jamurtas AZ, Nikolaidis MG, Michailidis I, Douroudos I, et al Time-course of changes in inflammatory and performance responses following a soccer game. Clinical journal of sport medicine: official journal of the Canadian Academy of Sport Medicine. 2008;18(5):423–31. Epub 2008/09/23. 18806550 10.1097/JSM.0b013e3181818e0b

[2] Halson SL. Monitoring training load to understand fatigue in athletes. Sports medicine. 2014;44 Suppl 2:S139–47. Epub 2014/09/10. 25200666 10.1007/s40279-014-0253-z PMC4213373

[3] Marqués-Jiménez D, Calleja-González J, Arratibel I, Delextrat A, Terrados N. Fatigue and Recovery in Soccer: Evidence and Challenges. The Open Sports Sciences Journal. 2017;10(Suppl 1: M5):52–70.

[4] Akenhead R, Nassis GP. Training Load and Player Monitoring in High-Level Football: Current Practice and Perceptions. International journal of sports physiology and performance. 2016;11(5):587–93. Epub 2015/10/13. doi: 10.1123/ijspp.2015-0331 26456711 10.1123/ijspp.2015-0331

[5] Swarup V, Rajeswari MR. Circulating (cell-free) nucleic acids—a promising, non-invasive tool for early detection of several human diseases. FEBS letters. 2007;581(5):795–9. Epub 2007/02/10. doi: 10.1016/j.febslet.2007.01.051 17289032 10.1016/j.febslet.2007.01.051

[6] Breitbach S, Tug S, Simon P. Circulating cell-free DNA: an up-coming molecular marker in exercise physiology. Sports medicine. 2012;42(7):565–86. Epub 2012/06/15. doi: 10.2165/11631380-000000000-00000 22694348 10.2165/11631380-000000000-00000

[7] Atamaniuk J, Vidotto C, Tschan H, Bachl N, Stuhlmeier KM, Muller MM. Increased concentrations of cell-free plasma DNA after exhaustive exercise. Clin Chem. 2004;50(9):1668–70. Epub 2004/08/28. doi: 10.1373/clinchem.2004.034553 15331502 10.1373/clinchem.2004.034553

[8] Atamaniuk J, Stuhlmeier KM, Vidotto C, Tschan H, Dossenbach-Glaninger A, Mueller MM. Effects of ultra-marathon on circulating DNA and mRNA expression of pro- and anti-apoptotic genes in mononuclear cells. Eur J Appl Physiol. 2008;104(4):711–7. Epub 2008/07/25. doi: 10.1007/s00421-008-0827-2 18651163 10.1007/s00421-008-0827-2

[9] Fatouros IG, Destouni A, Margonis K, Jamurtas AZ, Vrettou C, Kouretas D, et al Cell-free plasma DNA as a novel marker of aseptic inflammation severity related to exercise overtraining. Clinical chemistry. 2006;52(9):1820–4. Epub 2006/07/15. doi: 10.1373/clinchem.2006.070417 16840584 10.1373/clinchem.2006.070417

[10] Atamaniuk J, Vidotto C, Kinzlbauer M, Bachl N, Tiran B, Tschan H. Cell-free plasma DNA and purine nucleotide degradation markers following weightlifting exercise. European journal of applied physiology. 2010;110(4):695–701. Epub 2010/06/26. doi: 10.1007/s00421-010-1532-5 20577758 10.1007/s00421-010-1532-5

[11] Fatouros IG, Jamurtas AZ, Nikolaidis MG, Destouni A, Michailidis Y, Vrettou C, et al Time of sampling is crucial for measurement of cell-free plasma DNA following acute aseptic inflammation induced by exercise. Clin Biochem. 2010;43(16–17):1368–70. Epub 2010/08/31. doi: 10.1016/j.clinbiochem.2010.08.020 20800058 10.1016/j.clinbiochem.2010.08.020

[12] Beiter T, Fragasso A, Hudemann J, Niess AM, Simon P. Short-term treadmill running as a model for studying cell-free DNA kinetics in vivo. Clinical chemistry. 2011;57(4):633–6. Epub 2011/02/08. doi: 10.1373/clinchem.2010.158030 21296972 10.1373/clinchem.2010.158030

[13] Breitbach S, Sterzing B, Magallanes C, Tug S, Simon P. Direct measurement of cell-free DNA from serially collected capillary plasma during incremental exercise. Journal of applied physiology. 2014;117(2):119–30. Epub 2014/05/31. doi: 10.1152/japplphysiol.00002.2014 24876361 10.1152/japplphysiol.00002.2014

[14] Helmig S, Fruhbeis C, Kramer-Albers EM, Simon P, Tug S. Release of bulk cell free DNA during physical exercise occurs independent of extracellular vesicles. European journal of applied physiology. 2015;115(11):2271–80. Epub 2015/07/02. doi: 10.1007/s00421-015-3207-8 26126838 10.1007/s00421-015-3207-8

[15] Tug S, Mehdorn M, Helmig S, Breitbach S, Ehlert T, Simon P. Exploring the Potential of cfDNA Measurements After an Exhaustive Cycle Ergometer Test as a Marker for Performance Related Parameters. International journal of sports physiology and performance. 2016:1–24. Epub 2016/09/13. 27617485 10.1123/ijspp.2016-0157

[16] Velders M, Treff G, Machus K, Bosnyak E, Steinacker J, Schumann U. Exercise is a potent stimulus for enhancing circulating DNase activity. Clin Biochem. 2014;47(6):471–4. Epub 2014/01/01. doi: 10.1016/j.clinbiochem.2013.12.017 24373926 10.1016/j.clinbiochem.2013.12.017

[17] Haller N, Tug S, Breitbach S, Jorgensen A, Simon P. Increases in Circulating, Cell-Free DNA During Aerobic Running Depend on Intensity and Duration. International journal of sports physiology and performance. 2016:1–21. Epub 2016/09/13. 27617389 10.1123/ijspp.2015-0540

[18] Krustrup P, Mohr M, Steensberg A, Bencke J, Kjaer M, Bangsbo J. Muscle and blood metabolites during a soccer game: implications for sprint performance. Medicine and science in sports and exercise. 2006;38(6):1165–74. Epub 2006/06/16. doi: 10.1249/01.mss.0000222845.89262.cd 16775559 10.1249/01.mss.0000222845.89262.cd

[19] Fatouros IG, Chatzinikolaou A, Douroudos II, Nikolaidis MG, Kyparos A, Margonis K, et al Time-course of changes in oxidative stress and antioxidant status responses following a soccer game. Journal of strength and conditioning research / National Strength & Conditioning Association. 2010;24(12):3278–86. Epub 2009/12/10. 10.1519/JSC.0b013e3181b60444 19996787

[20] Breitbach S, Tug S, Helmig S, Zahn D, Kubiak T, Michal M, et al Direct quantification of cell-free, circulating DNA from unpurified plasma. PloS one. 2014;9(3):e87838 Epub 2014/03/07. doi: 10.1371/journal.pone.0087838 24595313 10.1371/journal.pone.0087838 PMC3940427

[21] Cunniffe B, Hore AJ, Whitcombe DM, Jones KP, Baker JS, Davies B. Time course of changes in immuneoendocrine markers following an international rugby game. European journal of applied physiology. 2010;108(1):113–22. Epub 2009/09/17. doi: 10.1007/s00421-009-1200-9 19756700 10.1007/s00421-009-1200-9

[22] Ascensao A, Rebelo A, Oliveira E, Marques F, Pereira L, Magalhaes J. Biochemical impact of a soccer match—analysis of oxidative stress and muscle damage markers throughout recovery. Clinical biochemistry. 2008;41(10–11):841–51. Epub 2008/05/07. doi: 10.1016/j.clinbiochem.2008.04.008 18457670 10.1016/j.clinbiochem.2008.04.008

[23] Chatzinikolaou A, Draganidis D, Avloniti A, Karipidis A, Jamurtas AZ, Skevaki CL, et al The microcycle of inflammation and performance changes after a basketball match. Journal of sports sciences. 2014;32(9):870–82. Epub 2014/02/01. doi: 10.1080/02640414.2013.865251 24479464 10.1080/02640414.2013.865251

[24] Andersson H, Bohn SK, Raastad T, Paulsen G, Blomhoff R, Kadi F. Differences in the inflammatory plasma cytokine response following two elite female soccer games separated by a 72-h recovery. Scandinavian journal of medicine & science in sports. 2010;20(5):740–7. Epub 2009/09/22. 19765242 10.1111/j.1600-0838.2009.00989.x

[25] Romagnoli M, Sanchis-Gomar F, Alis R, Risso-Ballester J, Bosio A, Graziani RL, et al Changes in muscle damage, inflammation, and fatigue-related parameters in young elite soccer players after a match. The Journal of sports medicine and physical fitness. 2016;56(10):1198–205. Epub 2016/11/04. 26558831

[26] Nedelec M, McCall A, Carling C, Legall F, Berthoin S, Dupont G. Recovery in soccer: part I—post-match fatigue and time course of recovery. Sports medicine. 2012;42(12):997–1015. Epub 2012/10/11. doi: 10.2165/11635270-000000000-00000 23046224 10.2165/11635270-000000000-00000

[27] Thorpe R, Sunderland C. Muscle Damage, Endocrine, and Immune Marker Response to a Soccer Match. Journal of Strength and Conditioning Research. 2012;26(10):2783–90. doi: 10.1519/JSC.0b013e318241e174 22124357 10.1519/JSC.0b013e318241e174

[28] Coutts AJ, Rampinini E, Marcora SM, Castagna C, Impellizzeri FM. Heart rate and blood lactate correlates of perceived exertion during small-sided soccer games. Journal of science and medicine in sport / Sports Medicine Australia. 2009;12(1):79–84. Epub 2007/12/11. 10.1016/j.jsams.2007.08.005 18068433

[29] Tug S, Helmig S, Deichmann ER, Schmeier-Jurchott A, Wagner E, Zimmermann T, et al Exercise-induced increases in cell free DNA in human plasma originate predominantly from cells of the haematopoietic lineage. Exercise immunology review. 2015;21:164–73. Epub 2015/04/01. 25826002

[30] Beiter T, Fragasso A, Hudemann J, Schild M, Steinacker J, Mooren FC, et al Neutrophils release extracellular DNA traps in response to exercise. Journal of applied physiology. 2014;117(3):325–33. doi: 10.1152/japplphysiol.00173.2014 24833781 10.1152/japplphysiol.00173.2014

[31] Villa F. Detection of Circulating Tumor DNA Moves Closer To Clinical Use. Prevention and Infection Control. 2015.

[32] Evans CJ, Aguilera RJ. DNase II: genes, enzymes and function. Gene. 2003;322:1–15. Epub 2003/12/04. 14644493 10.1016/j.gene.2003.08.022

[33] Hermansen L, Osnes JB. Blood and muscle pH after maximal exercise in man. J Appl Physiol. 1972;32(3):304–8. Epub 1972/03/01. doi: 10.1152/jappl.1972.32.3.304 5010039 10.1152/jappl.1972.32.3.304

[34] Osnes JB, Hermansen L. Acid-base balance after maximal exercise of short duration. J Appl Physiol. 1972;32(1):59–63. Epub 1972/01/01. doi: 10.1152/jappl.1972.32.1.59 5007019 10.1152/jappl.1972.32.1.59

